# Propionic acid disrupts endocytosis, cell cycle, and cellular respiration in yeast

**DOI:** 10.1186/s13104-021-05752-z

**Published:** 2021-08-28

**Authors:** Emma W. Micalizzi, Ashkan Golshani, Myron L. Smith

**Affiliations:** Department of Biology, Carleton University, Ottawa, ON Canada

**Keywords:** Propionic acid, Endocytosis, Cell cycle, Cellular respiration, Volatile antifungal, *Saccharomyces cerevisiae*

## Abstract

**Objective:**

We previously identified propionic acid as a microbially-produced volatile organic compound with fungicidal activity against several pathogenic fungi. The purpose of this work is to better understand how propionic acid affects fungi by examining some of the effects of this compound on the yeast cell.

**Results:**

We show that propionic acid causes a dramatic increase in the uptake of lucifer yellow in yeast cells, which is consistent with enhanced endocytosis. Additionally, using a propidium iodide assay, we show that propionic acid treatment causes a significant increase in the proportion of yeast cells in G_1_ and a significant decrease in the proportion of cells in G_2_, suggesting that propionic acid causes a cell cycle arrest in yeast. Finally, we show that the reduction of MTT is attenuated in yeast cells treated with propionic acid, indicating that propionic acid disrupts cellular respiration. Understanding the effects of propionic acid on the yeast cell may aid in assessing the broader utility of this compound.

**Supplementary Information:**

The online version contains supplementary material available at 10.1186/s13104-021-05752-z.

## Introduction

Propionic acid is a weak organic acid that is widely used as a food preservative and generally considered to be safe by regulatory bodies in Canada [1], the United States [2], and the European Union [3]. Propionic acid can be produced by microbes and is fungicidal towards the bat pathogen *Pseudogymnoascus destructans* [4] and several plant pathogenic fungi (Additional file 1: Figure S1). A volatile organic compound like propionic acid holds promise as a fumigant for controlling fungal pathogens in agricultural soils [e.g. 5], bat hibernacula [6], and other complex and textured environments.

In this work, we examine effects of propionic acid on baker’s yeast (*Saccharomyces cerevisiae*) to better understand potential applications for this compound. We examined pathways that were enriched in chemical-genetic profiles amongst highly sensitive deletion mutants (unpublished observations) and show that propionic acid affects evolutionarily conserved processes in yeast including endocytosis, the cell cycle, and cellular respiration.

## Main text

All assays used *S. cerevisiae* strain S288C and sub-inhibitory concentrations of liquid propionic acid. To ensure that the observed effects of propionic acid were not strictly due to growth inhibition, we used the antifungal aldehyde nonanal as a positive control. For both propionic acid and nonanal, a sub-inhibitory concentration (4.7 μl ml^−1^ and 0.5 μl ml^−1^, respectively) was selected that reduced yeast cell survival by 20% compared to the no-treatment control. For each mode of action assay, technical replicates were averaged and mean values of each biological replicate were used for statistical analysis in R [7].

### Propionic acid exposure increases endocytosis in yeast cells

We examined yeast cells exposed to propionic acid for uptake of lucifer yellow, a hydrophilic fluorescent dye that enters yeast cells by endocytosis [8–10]. *S. cerevisiae* was grown overnight in YPD (yeast, peptone, d-glucose) media and then adjusted to an OD_600_ of 0.80 before 100 µl aliquots of cell suspension were mixed with 100 µl buffer (12.5 mM sodium phosphate, 2.5 mM sodium fluoride) and treated with 0.95 µl propionic acid, 0.1 µl nonanal, or a no-treatment control. Cell suspensions were then incubated at 30 °C for 30 min before adding lucifer yellow to a final concentration of 4 mg ml^−1^ and incubating at 30 °C for an additional 3 h. Cells were then washed three times and resuspended in buffer before measuring fluorescence using a BD Accuri C6 flow cytometer. Three independent experiments were performed, each with three replicates and a minimum of 10,000 cells counted per replicate. Cells were also photographed using a Zeiss Axioplan 2 imaging microscope with an AxioCam HRm camera.

**Fig. 1:**
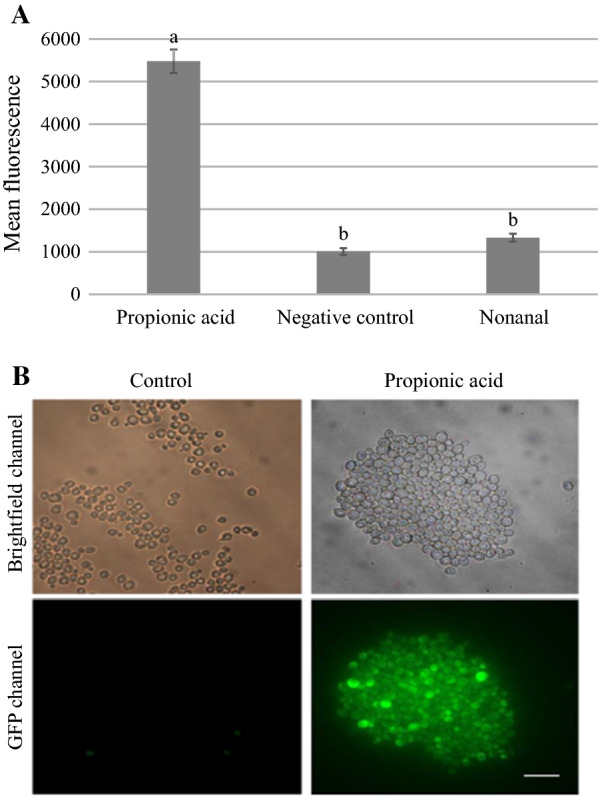
Lucifer yellow uptake in yeast cells exposed to propionic acid. Yeast cells were treated with propionic acid, nonanal, or a carrier control in the presence of lucifer yellow. **A** shows the mean fluorescence of yeast cells with standard error bars (n = 3). Different letters indicate significant differences (p < 0.001). **B** shows micrographs in brightfield (top) and fluorescence (bottom) channels that contrast lucifer yellow uptake in yeast cells in a no-treatment negative control (left) compared to treatment with propionic acid (right). The white scale bar represents 25 µm

There was a significant effect of compound treatment on cell fluorescence (one-way ANOVA, F(2,6) = 136.36, p < 0.001; Fig. 1A). Lucifer yellow fluorescence of cells treated with propionic acid was significantly greater than that of cells treated with nonanal or the negative control (Tukey test, p < 0.001 for both comparisons), while the fluorescence of cells in the nonanal and negative control treatments did not differ significantly (Tukey test, p = 0.56). This indicates that propionic acid increases endocytosis in yeast cells, and this was further supported by fluorescence microscopy (Fig. 1B).

Increased endocytosis may be a response to membrane damage with exposure to weak acids such as propionic acid [11]. Damaged membrane proteins can activate quality control mechanisms that cause their endocytosis and degradation in the multivesicular body pathway [12–15]. Interestingly, genes involved in protein catabolism through the multivesicular body pathway contribute to propionic acid resistance [16], suggesting that endocytosis of damaged surface proteins may be part of an adaptive response to propionic acid.

### Propionic acid disrupts the yeast cell cycle

We assessed if propionic acid affects cell cycle progression by staining and measuring DNA content, and then calculating the percentage of cells in G_1_, S, and G_2_ phases [17]. Yeast cells were grown overnight as described above before 200 µl aliquots of cell suspension were pipetted into 1.5 ml epitubes with 0.95 µl propionic acid, 0.1 µl nonanal, or a no-compound control, and incubated at 30 °C for 3 h. Cells were then pelleted by centrifugation and fixed by resuspending in 500 µl of 70% ethanol. Cells were then incubated at 22 °C for 2.5 h and resuspended in 500 µl phosphate-buffered saline (PBS) for 10 min before pelleting and resuspending in 100 µl PBS with 1 mg ml^−1^ RNase A. Cells were incubated overnight at 37 °C and then pelleted and resuspended in 100 µl PBS with 50 µg ml^−1^ propidium iodide before incubating in the dark at 37 °C for 1 h. Propidium iodide staining was analysed using a BD Accuri C6 flow cytometer and the percentage of cells in each phase of the cell cycle was calculated using ModFit LT (Verity Software House, Topsham, Maine). Three independent experiments were conducted, each with three replicates and 10,000 cells counted per replicate.

**Fig. 2:**
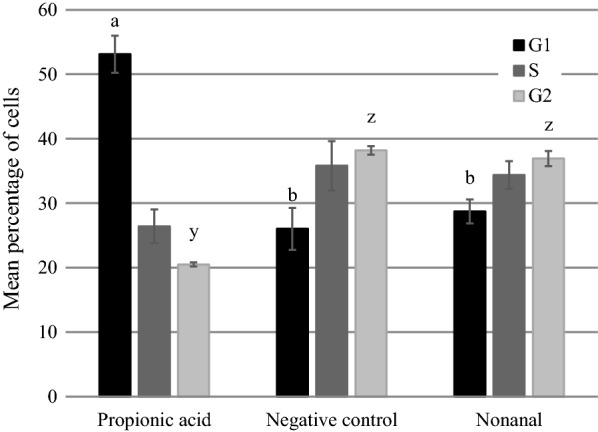
Cell cycle distributions in yeast cells treated with propionic acid. Cells were exposed to propionic acid, nonanal, or a no-treatment control before staining with propidium iodide to assess DNA content by flow cytometry. Percentage of cells in each cell cycle phase was calculated using ModFit LT. Error bars show standard error (n = 3) and letters show significant differences (p < 0.01) for the proportion of cells in G1 (a and b) and G2 (y and z)

As illustrated in Fig. 2, one-way ANOVAs conducted for each cell cycle phase showed a significant effect of compound treatment on the percentage of cells in G_1_ (F(2,6) = 19.91; p = 0.002) and G_2_ (F(2,6) = 101.60; p < 0.001), but not S-phase (F(2,6) = 1.97; p = 0.22). Specifically, post-hoc Tukey tests showed that compared to nonanal-treated cells and negative control cells, propionic acid-treated cells had a greater percentage of cells in G_1_ (p < 0.003) and a lower percentage of cells in G_2_ (p < 0.001), whereas the percentage of cells in S-phase was not significantly different for any treatments (p > 0.2). The percentage of cells in each phase of the cell cycle did not differ significantly between the nonanal and negative control treatments (p > 0.65 for all comparisons).

Together, these results indicate that propionic acid causes a G_1_ or S-phase arrest, which are difficult to distinguish without detailed examination [18]. G_1_ or S-phase arrests can be due to small cell size, DNA damage, and DNA replication stress [19–21], as well as membrane permeabilization [22]. To our knowledge, this is the first report indicating that propionic acid perturbs the cell cycle in fungi.

### Propionic acid inhibits cellular respiration

We tested if propionic acid affects cellular respiration by measuring the reduction of MTT (3-(4,5-dimethyl-2-thiazolyl)-2,5-diphenyl-2*H*-tetrazolium bromide) [23, 24], which is converted to a purple formazan salt by NADH-dependent reactions in metabolically active cells [25]. Yeast cells were grown overnight in YPD, washed twice, and resuspended in sterile distilled water. Cells were incubated at 22 °C for approximately 10 h, resuspended at an OD_600_ of 0.80 in YPD and 150 µl aliquots were placed into a microtiter plate with 15 µl of 2.89 mM MTT, 10 µl 0.19 mM phenazine methosulfate, and 25 µl 10% Triton X-100, before adding 0.95 µl propionic acid, 0.1 µl nonanal, or a carrier control. The microtiter plate was sealed with Parafilm and placed into a BioTek Instruments Cytation 5 microtiter plate reader set to 30 °C with continuous shaking (282 double orbital cycles per minute). We conducted three independent experimental replicates, each with technical replicates comprising two cell-free controls, five propionic acid treatments, five nonanal growth inhibition controls, and five no-inhibitor controls. Absorbance values at 570 and 660 nm were measured every 5 min for 9 h to account for MTT absorbance and cell growth, respectively. To normalize for growth and compound absorbance, an MTT reduction score was calculated at each time point as $\frac{{A_{570} \exp . - A_{570} cont.}}{{A_{660} \exp .}}$ where A_570_ exp. and A_660_ exp. are the absorbances of the cell suspension, and A_570_ cont. is the absorbance of the cell-free suspension.

**Fig. 3:**
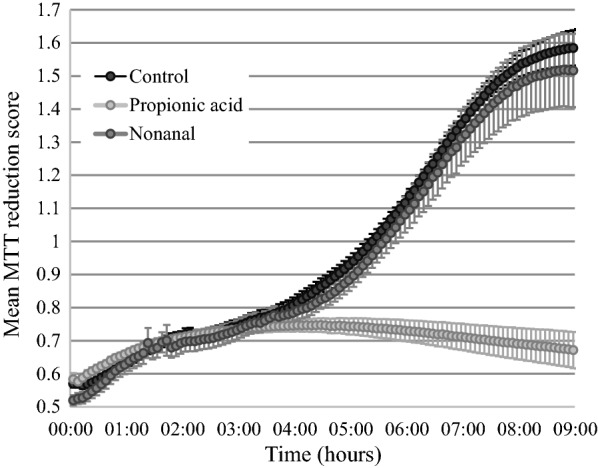
Effect of propionic acid on yeast reduction of MTT. Yeast cells were exposed to propionic acid, nonanal, or a no-compound control, and the reduction of MTT was monitored spectrophotometrically for 9 h. Mean MTT reduction scores are plotted with standard error bars (n = 3)

There was a significant effect of compound treatment on the reduction of MTT at 9 h (one-way ANOVA, F(2,6) = 7.50, p = 0.02; Fig. 3), such that the MTT reduction score in cells treated with propionic acid was significantly lower than in cells in the no-treatment control (Tukey test, p = 0.03) and the nonanal control (Tukey test, p = 0.05). The reduction of MTT in the no-treatment control and nonanal growth-inhibition control was very similar in both endpoint reduction (Tukey test, p = 0.92) and kinetics, suggesting that the effects of propionic acid are not simply due to growth inhibition. The kinetics of the MTT assay with propionic acid suggest that this compound has an inhibitory effect within 2–3 h of exposure, consistent with our previous accounts of time for propionic acid to inhibit *P. destructans*, the causal agent of bat white-nose syndrome [6].

Propionic acid has been shown to indirectly inhibit the pyruvate dehydrogenase complex [26], which links glycolysis and the citric acid cycle by converting pyruvate into acetyl-CoA. Our results are consistent with this, as inhibition of pyruvate dehydrogenase would decrease the generation of NADH necessary to reduce MTT. However, it is worth noting that the reduction of MTT in the propionic acid no-cell control was lower than in all other no-cell conditions (unpublished observations). This was accounted for in the calculated MTT reduction score, but it suggests that propionic acid interferes with non-enzymatic background reduction of MTT.

## Conclusions

In this work, we used a yeast model to show that exposure to sub-inhibitory concentrations of propionic acid leads to a dramatic increase in endocytosis, changes to the cell cycle, and a disruption of cellular respiration. Previous research has shown that propionic acid also affects membrane permeability and acidifies the cytoplasm [27–29]; but see [16], causes oxidative stress and apoptosis [30]; but see [31], and affects glucose metabolism [26]. This range of effects of propionic acid on the cell suggests that the compound may target a central regulator of cellular homeostasis, or several cellular processes simultaneously.

Further understanding the cellular effects of propionic acid may be useful in identifying other applications and predicting off-target effects. For example, our results suggest that subinhibitory concentrations of propionic acid could be used as an inducer of endocytosis for research purposes or to enhance uptake (synergist) of other drugs. Considering off-target effects, propionic acid is generally considered to be safe for human use [1–3] and has low acute oral toxicity in rats (LD_50_ 351–3470 mg kg^−1^ body weight) and dogs (LD_50_ > 1832 mg kg^−1^ body weight with repeated dietary exposure); exposure generally causes inflammation and irritation, rather than systemic toxicity [32]. Nevertheless, we show that the compound clearly disrupts the yeast cell cycle, which is highly conserved among eukaryotes. This adds to evidence from studies with plants [e.g. 33] and human cell cultures [e.g. 34] that propionic acid causes cell cycle arrest.

## Limitations

Future research should test whether the observed effects of propionic acid on yeast also occur in other fungi and eukaryotes in general. Clearly, the biochemical processes that we examined are largely conserved, and suggest that off-target effects could occur with this common food preservative. Future research should further build upon our observations to determine the mechanism(s) of action of propionic acid.

## Supplementary Information

**Additional file 1: Figure S1.** Growth inhibition of plant pathogenic fungi by volatile propionic acid. Approximately 50 CFU of plant pathogenic fungi were inoculated onto PDA plates and incubated until the initiation of exponential growth. Cultures were then exposed to the volatile phase from 25 μl propionic acid (green), or a no-volatile control (purple), for 4 h before propionic acid was removed. The time of fumigation is shown by the red arrows, after which colony diameter was measured daily for 1 week.

**Additional file 2.**  Spreadsheet containing all raw data generated in our experiments and used for analysis in this manuscript.

## Data Availability

All data generated or analysed during this study are included in this published article and its Additional files (Additional file 2).

## Abbreviations

MTT: 3-(4,5-Dimethyl-2-thiazolyl)-2,5-diphenyl-2*H*-tetrazolium bromide
YPD: Yeast, peptone, d-glucose media

## References

[1] Environment and Climate Change Canada and Health Canada. Screening assessment carboxylic acids group. 2019. https://www.canada.ca/en/environment-climate-change/services/evaluating-existing-substances/screening-assessment-carboxylic-acids-group.html. Accessed 7 Jan 2021.

[2] Food and Drug Administration. Sec. 184.1081 propionic acid. Title 21, Volume 3, Chapter 1, Part 184. 2017. https://www.accessdata.fda.gov/scripts/cdrh/cfdocs/cfcfr/CFRSearch.cfm?fr=184.1081. Accessed 20 Nov 2017.

[3] European Food Safety Authority (2014). Scientific opinion on the re-evaluation of propionic acid (E 280), sodium propionate (E 281), calcium propionate (E 282) and potassium propionate (E 283) as food additives. EFSA J.

[4] Micalizzi EW, Mack JN, White GP, Avis TJ, Smith ML (2017). Microbial inhibitors of the fungus *Pseudogymnoascus destructans*, the causal agent of white-nose syndrome in bats. PLoS ONE.

[5] Stinson AM, Zidack NK, Strobel GA, Jacobson BJ (2003). Mycofumigation with *Muscodor albus* and *Muscodor roseus* for control of seedling diseases of sugar beet and verticillium wilt of eggplant. Plant Dis.

[6] Micalizzi EW, Smith ML (2020). Volatile organic compounds kill the white-nose syndrome fungus, *Pseudogymnoascus destructans*, in hibernaculum sediment. Can J Microbiol.

[7] R Core Team. R: a language and environment for statistical computing. R Foundation for Statistical Computing. 2017. https://www.R-project.org/.

[8] Dulic V, Egeron M, Elguindi I, Raths S, Singer B, Riezman H (1991). Yeast endocytosis assays. Methods Enzymol.

[9] Wiederkehr A, Meier KD, Riezman H (2001). Identification and characterization of *Saccharomyces cerevisiae* mutants defective in fluid-phase endocytosis. Yeast.

[10] Motizuki M, Yokota S, Tsurugi K (2008). Effect of low pH on organization of the actin cytoskeleton in *Saccharomyces cerevisiae*. Biochim Biophys Acta.

[11] Mira NP, Teixeira MC, Sá-Correia I (2010). Adaptive response and tolerance to weak acids in *Saccharomyces cerevisiae*: a genome-wide view. OMICS.

[12] Feyder S, De Craene J-O, Bär S, Bertazzi DL, Friant S (2015). Membrane trafficking in the yeast *Saccharomyces cerevisiae* model. Int J Mol Sci.

[13] Goode BL, Eskin JA, Wendland B (2015). Actin and endocytosis in budding yeast. Genetics.

[14] Babst M (2014). Quality control at the plasma membrane: one mechanism does not fit all. J Cell Biol.

[15] Li Y, Kane T, Tipper C, Spatrick P, Jenness DD (1999). Yeast mutants affecting possible quality control of plasma membrane proteins. Mol Cell Biol.

[16] Ullah A, Orij R, Brul S, Smits GJ (2012). Quantitative analysis of the modes of growth inhibition by weak organic acids in *Saccharomyces cerevisiae*. Appl Environ Microbiol.

[17] Wu X, Liu L, Huang M (2011). Checkpoints studies using the budding yeast *Saccharomyces cerevisiae*. Methods Mol Biol.

[18] Dolbeare F, Gratzner H, Pallavicini MG, Gray JW (1983). Flow cytometric measurement of total DNA content and incorporated bromodeoxyuridine. Proc Natl Acad Sci USA.

[19] Bertoli C, Skotheim JM, de Bruin RAM (2013). Control of cell cycle transcription during G1 and S phases. Nat Rev Mol Cell Biol.

[20] Gerald JN, Benjamin JM, Kron SJ (2002). Robust G1 checkpoint arrest in budding yeast: dependence on DNA damage signalling and repair. J Cell Sci.

[21] Barnum KJ, O’Connell MJ (2014). Cell cycle regulation by checkpoints. Methods Mol Biol.

[22] Kono K, Al-Zain A, Schroeder L, Nakanishi M, Ikui AE (2016). Plasma membrane/cell wall perturbation activates a novel cell cycle checkpoint during G1 in *Saccharomyces cerevisiae*. Proc Natl Acad Sci USA.

[23] Stowe RP, Koenig DW, Mishra SK, Pierson DL (1995). Nondestructive and continuous spectrophotometric measurement of cell respiration using a tetrazolium-formazan microemulsion. J Microbiol Methods.

[24] Sánchez SN, Königsberg M (2006). Using yeast to easily determine mitochondrial functionality with 1-(4,5-dimethylthiazol-2-yl)-3,5-diphenyltetrazolium bromide (MTT) assay. Biochem Mol Biol Educ.

[25] Riss TL, Moravec RA, Niles AL, Duellman S, Benink HA, Worzella TJ, et al. Cell viability assays. Assay guidance manual. 2016. https://www.ncbi.nlm.nih.gov/books/NBK144065. Accessed 26 Nov 2017.

[26] Brock M, Buckel W (2004). On the mechanism of action of the antifungal agent propionate. FEBS J.

[27] Ahmadi N, Khosravi-Darani K, Mortazavian AM (2016). An overview of biotechnological production of propionic acid: from upstream to downstream processes. Electron J Biotechnol.

[28] Ferreira MM, Loureiro-Dias MC, Loureiro V (1997). Weak acid inhibition of fermentation by *Zygosaccharomyces bailii* and *Saccharomyces cerevisiae*. Int J Food Microbiol.

[29] Davidson PM, Juneja VK, Branen JK, Branen AL, Davidson PM, Saliminen S, Thorngate JH (2002). Antimicrobial Agents. Food additives.

[30] Yun J, Lee DG (2016). A novel fungal killing mechanism of propionic acid. FEMS Yeast Res.

[31] Semchyshyn HM, Abrat OB, Miedzobrodzki J, Inoue Y, Lushchak VI (2011). Acetate but not propionate induced oxidative stress in bakers’ yeast *Saccharomyces cerevisiae*. Redox Rep.

[32] Organisation for Economic Co-operation and Development. CAS No. 79-09-4. SIDS initial assessment report: propionic acid. 2007. https://hpvchemicals.oecd.org/UI/handler.axd?id=6ccb362f-dcec-4a69-b6eb-730e90edb94f. Accessed 20 Jan 2021.

[33] Tramontano WA, DeLillo AR, Yung SY, Natarajan C, Kearns CM (1991). Short-chain fatty-acid-induced effects on the cell cycle in root meristems of *Pisum sativum*. Physiol Plant.

[34] Kim K, Kwon O, Ryu TY, Jung C-R, Kim J, Min J-K (2019). Propionate of a microbiota metabolite induces cell apoptosis and cell cycle arrest in lung cancer. Mol Med Rep.

