# Supplementary material for: Propionic acid disrupts endocytosis, cell cycle, and cellular respiration in yeast
Source: BMC Res Notes. 2021 Aug 28;14:335. doi: 10.1186/s13104-021-05752-z (PMC8403364; doi:10.1186/s13104-021-05752-z)
Supplement: Supplementary file 1 — Additional file 1: Figure S1. Growth inhibition of plant pathogenic fungi by volatile propionic acid. Approximately 50 CFU of plant pathogenic fungi were inoculated onto PDA plates and incubated until the initiation of exponential growth. Cultures were then exposed to the volatile phase from 25 μl propionic acid (green), or a no-volatile control (purple), for 4 h before propionic acid was removed. The time of fumigation is shown by the red arrows, after which colony diameter was measured daily for 1 week. [file 13104_2021_5752_MOESM1_ESM.pptx]

## Slide 1
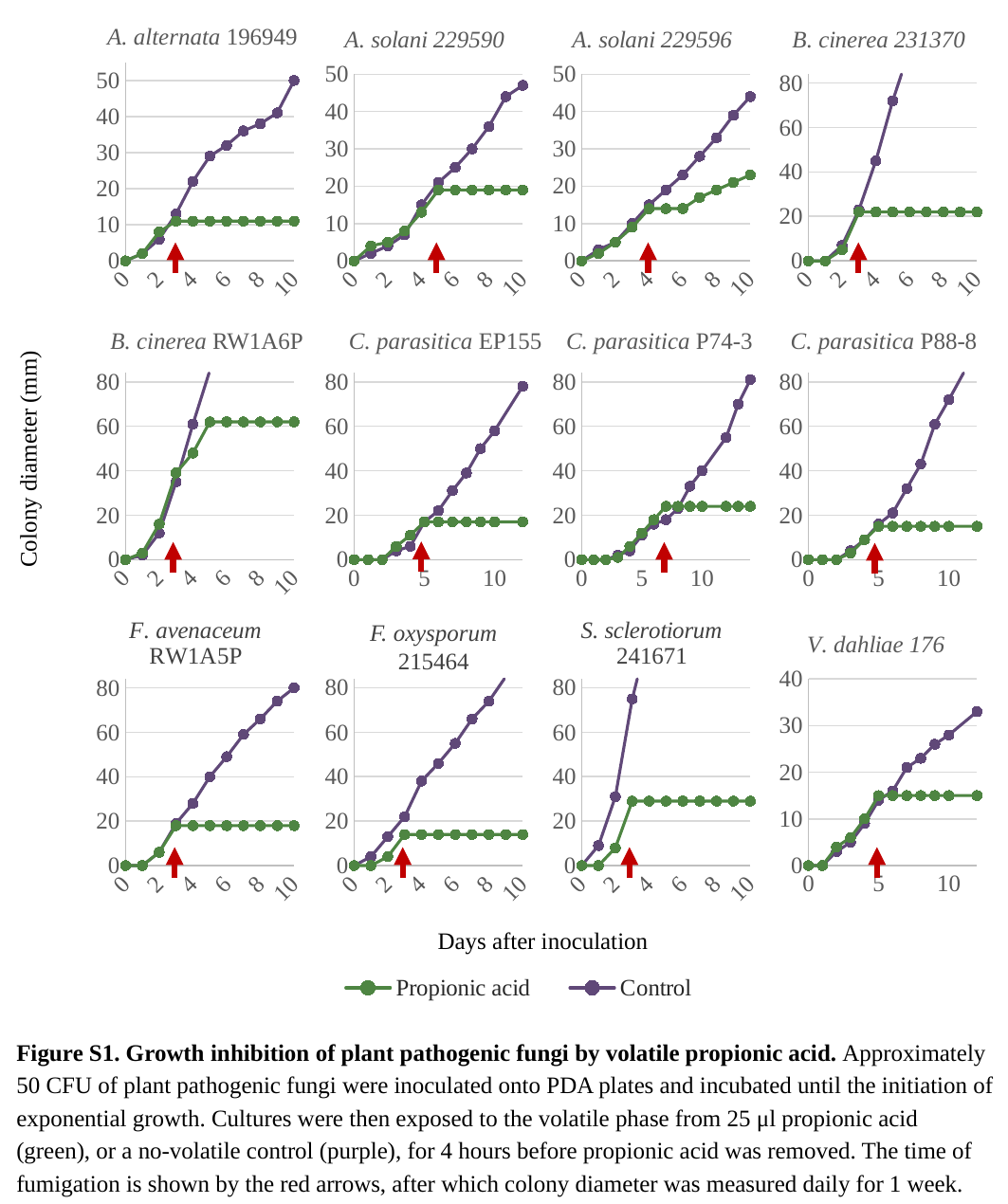

### Chart
| Category | Propionic acid | Control |
|---|---|---|
### Chart: A. solani 229590
| Category | Propionic acid | Control |
|---|---|---|
### Chart: A. solani 229596
| Category | Propionic acid | Control |
|---|---|---|
### Chart: B. cinerea 231370
| Category | Propionic acid | Control |
|---|---|---|A. alternata 196949
### Chart:
| Category | Propionic acid | Control |
|---|---|---|
### Chart
| Category | Propionic acid | Control |
|---|---|---|
### Chart:
| Category | Propionic acid | Control |
|---|---|---|
### Chart:
| Category | Propionic acid | Control |
|---|---|---|B. cinerea RW1A6P
C. parasitica EP155
C. parasitica P74-3
C. parasitica P88-8
Colony diameter (mm)
F. oxysporum 215464
### Chart:
| Category | Propionic acid | Control |
|---|---|---|
### Chart:
| Category | Propionic acid | Control |
|---|---|---|
### Chart:
| Category | Propionic acid | Control |
|---|---|---|
### Chart: V. dahliae 176
| Category | Propionic acid | Control |
|---|---|---|
### Chart
| Category | Propionic acid | Control |
|---|---|---|Days after inoculation
Figure S1. Growth inhibition of plant pathogenic fungi by volatile propionic acid. Approximately 50 CFU of plant pathogenic fungi were inoculated onto PDA plates and incubated until the initiation of exponential growth. Cultures were then exposed to the volatile phase from 25 μl propionic acid (green), or a no-volatile control (purple), for 4 hours before propionic acid was removed. The time of fumigation is shown by the red arrows, after which colony diameter was measured daily for 1 week.
